# Etiological and associated factors of thin endometrium among infertile women

**DOI:** 10.6026/973206300212622

**Published:** 2025-08-31

**Authors:** Kanchan Rani, Riya Goyal, Twisha Jain, Sakshi Jain, Priya Saxena

**Affiliations:** 1 Department of Obstetrics and Gynaecology, Teerthanker Mahaveer Medical College & Research Centre, Moradabad, Uttar Pradesh, India

**Keywords:** Chronic endometritis, thin endometrium, uterine fibroids, uterine curettage, genital tuberculosis

## Abstract

Thin endometrium is a condition that adversely affects infertility treatment outcomes by impairing embryo implantation and pregnancy success. Therefore, it is of interest to assess the etiological and associated factors of thin endometrium in 61 women undergoing infertility treatment. Thin endometrium was defined as ≤7 mm on transvaginal ultrasound. Tuberculosis was the most common cause (18 cases), followed by repeated curettage (12 cases), while 26 cases were unexplained. Advancing age (45.9%) and parity (22.9%) were the most common associated factors. Thus, we show that tuberculosis and endometrial trauma as major contributors to thin endometrium.

## Background:

A robust endometrial growth pattern is crucial for successful implantation. Ultrasonography often identifies patients with reduced endometrial thickness [1]. Numerous studies have associated low implantation rates with a "thin" endometrium, which is recognized as a critical factor in implantation failure [2]. Thin endometrium (TE) is defined as an endometrial thickness of ≤7 mm on ultrasonography [3, 4]. Enhancing endometrial development in women with TE remains challenging, despite several interventions including low-dose aspirin and estrogen therapy [3]. Irregularities in endometrial growth have long been considered a key cause of thin endometrium. An essential component of assisted reproduction is the evaluation of the endometrium, as endometrial thickness is a predictor of success [5, 6]. When the uterine lining is classified as "thin," both patients and clinicians face the difficult decision of whether to continue with the treatment cycle. However, there remains insufficient understanding of the factors contributing to diminished endometrial growth in women with thin endometrium [7]. Therefore, it is of interest to evaluate the etiological aspects of thin endometrium.

## Methodology:

It was a retrospective study at a tertiary care and IVF centre. After considering the utility of the study and obtaining approval from the ethical review committee, 61 patients of infertility with thin endometrium from January 2019 to December 20204 were enrolled for the study. Data such as name, age, etc., was recorded. The ultrasonographic definition was a maximal endometrial thickness of no more than 7 mm, as measured by transvaginal ultrasound scans before ovulation or after the administration of human chorionic gonadotropin (hCG). The causes of thin endometrium were recorded. The results were compiled and subjected to statistical analysis using the Mann-Whitney U test. A p-value less than 0.05 were regarded as significant.

## Results:

This study included 61 female patients with infertility, where the ultrasonographic measurement defined a thin endometrium as having a thickness of no more than 7 mm. The causes of thin endometrium were recorded and analyzed. The average age of the participants was 34.5 years, and the mean BMI was 23.8 kg/m^2^. The mean parity was 1.2, indicating most participants had one previous pregnancy. The average duration of infertility was 4.5 years, highlighting a prolonged period of infertility among the participants (Table 1 - see PDF). The most common cause of thin endometrium was tuberculosis, observed in 18 cases, followed by a history of repeated curettage (12 cases). A significant portion of the cases (21) had no identifiable cause, categorized as unexplained (Table 2 - see PDF). Advancing age was the most commonly associated factor present in 28 cases, followed by parity (14 cases) (Table 3 - see PDF). The hormonal profile of the participants showed an average TSH level of 2.8 mIU/L, a Prolactin level of 16.3 ng/mL, an LH level of 6.1 mIU/mL, and an FSH level of 7.9 mIU/mL. These values indicate a range within normal limits for most participants, though individual variations might contribute to the etiology of thin endometrium (Table 4 - see PDF). The mean uterine artery (UA) resistance index (RI) was 0.92, and the mean radial artery (RA) resistance index (RI) was 0.84 (Table 5 - see PDF).

## Discussion:

Endometrial receptivity is fundamental to implantation and pregnancy success in both natural and ART cycles [8]. Currently, there are no standardized criteria to evaluate endometrial receptivity in IVF patients [9]. Several ultrasonographic parameters-including thickness, echogenicity and pattern-have been assessed for their potential to predict implantation and pregnancy outcomes [10, 11]. Despite abundant literature, the predictive reliability of endometrial thickness remains debated, although some studies have noted a positive association between endometrial thickness and pregnancy rates [12, 13]. The present study aimed to explore the etiological factors linked with thin endometrium. Shufaro *et al.* [14] reported that 13 of 1,405 IVF patients repeatedly exhibited unresponsive thin endometrium (<7 mm), with 10 of them having a history of curettage-comparable to our observation of 19.6% with prior curettage. Similarly, Liu *et al.* highlighted that intrauterine surgeries such as D & C and adhesion lysis were leading causes of thin endometrium [15]. In our cohort, 29.5% had a history of genital tuberculosis, aligning with Sharma *et al.*'s finding of TB history in 67.8% of women with thin endometrium [16]. Advancing age was the most frequently associated factor in our study (28 cases), followed by parity (14 cases), while uterine fibroids and endometriosis were each found in 4 cases. The mean uterine artery resistance index (UA-RI) was 0.92±0.18, and the radial artery resistance index (RA-RI) was 0.84±0.12, both of which were elevated. These findings correspond with previous studies showing significantly increased RA-RI in patients with thin endometrium across the menstrual cycle, suggesting the role of vascular resistance and hormonal alterations [17]. Pulsed-wave Doppler studies further support that high uterine artery resistance is associated with adverse ART outcomes [18]. Dain *et al.* reported no statistically significant difference in CPR or LVBR between groups with endometrial thickness <6 mm and those with greater thickness, but observed more live births with 8.2 mm cut-off [19]. However, an Endometrial thickness < 8.2 mm was linked to lower live birth rates. A thin endometrium may fail to support implantation and fetal growth, leading to increased miscarriage and intrauterine death. Notably, patients with <6 mm Endometrial thickness had a higher prevalence of Grade A endometrial patterns and fewer Grade C patterns, indicating altered endometrial morphology.

## Conclusion:

The most common cause of thin endometrium was tuberculosis, followed by endometrial curettage and chronic endometritis. A large number of cases were idiopathic.

## References

[1] https://www.fertstert.org/.

[2] Strohmer H (1994). Fertil Steril..

[3] Scioscia M (2009). Reprod Biomed Online..

[4] Wang Y (2024). Front Endocrinol (Lausanne)..

[5] Miwa I (2009). Fertil Steril..

[6] Xu B (2015). Reprod Biomed Online..

[7] Mahajan N, Sharma S (2016). J Hum Reprod Sci..

[8] Senturk LM, Erel CT (2008). Curr Opin Obstet Gynecol..

[9] Barash A (2003). Fertil Steril..

[10] Raziel A (2007). Fertil Steril..

[11] Friedler S (1996). Hum Reprod Update..

[12] Sundstrom P (1998). Hum Reprod..

[13] Rijnders PM, Jansen CA (1998). Hum Reprod..

[14] Shufaro Y (2008). J Assist Reprod Genet..

[15] Liu KE (2019). Reprod Biomed Online..

[16] Sharma JB (2008). Arch Gynecol Obstet..

[17] Miwa I (2009). Fertil Steril..

[18] Raine-Fenning N (2008). Ultrasound Obstet Gynecol..

[19] Dain L (2013). Fertil Steril..

